# Selective Extraction of Gardenia Yellow and Geniposide from *Gardenia jasminoides* by Mechanochemistry

**DOI:** 10.3390/molecules21050540

**Published:** 2016-04-28

**Authors:** Wenhao Xu, Jingbo Yu, Wen Feng, Weike Su

**Affiliations:** 1 National Engineering Research Center for Process Development of Active Pharmaceutical Ingredients, Collaborative Innovation Center of Yangtze River Delta Region Green Pharmaceuticals, Zhejiang University of Technology, Hangzhou 310014, China; 2005101219990701@163.com (W.X.); yjb@zjut.edu.cn (J.Y.); fwmolly@zjut.edu.cn (W.F.); 2 Key Laboratory for Green Pharmaceutical Technologies and Related Equipment of Ministry of Education, College of Pharmaceutical Sciences, Zhejiang University of Technology, Hangzhou 310014, China

**Keywords:** *Gardenia jasminoides*, gardenia yellow, geniposide, mechanochemistry, selective extraction

## Abstract

A novel method for the selective extraction of gardenia yellow and geniposide from *Gardenia Jasminoides*, based on a mechanochemical method is described. Without the need of complex separation techniques, gardenia yellow compliant with the national standard could be extracted in a simple fashion. The optimal ball-milling conditions determined were as follows: 30% g/g. active carbon milling at 200 rpm in a planetary mill for 5 min. The extraction conditions of the milled mixtures were as follows: the milled mixtures were extracted with water (liquid-solid ratio 10:1) at 20 °C for 5 min with yields 85% of total geniposide, followed by extraction with 80% ethanol solution (liquid-solid ratio 5:1) and 1% g/g. Tween 20 at 75 °C for 5 min to yield 1.45% ± 0.108% g/g of gardenia yellow. The mechanism of this selective extraction was demonstrated to follow a microstructure change of activated carbon, which occurred during milling and lead to alteration of the corresponding desorption capacities. Compared with traditional extraction methods, this novel extraction technique greatly simplifies the separation process, and proves to be advantageous in terms of low organic solvent consumption, easy operation, rapid process and high efficiency.

## 1. Introduction

Gardenia (*Gardenia jasminoides*) is an evergreen shrub of the family *Rubiaceae* of which the primary medically relevant part is the fruit. In traditional Chinese medicine, gardenia is used individually to cure diseases including angina pectoris [1] and jaundice [2]. Furthermore, gardenia can also be used as an economically feasible plant for the preparation of various health supplements. To date, almost all active compounds in gardenia have been separated and individually identified. According to their chemical structures, these compounds can be classified into four types, namely iridoids, carotenoids, flavonoids and terpenes [3]. Among those identified types, the most abundant are carotenoids and iridoids [4].

The water-soluble carotenoid fraction in gardenia, which mainly consists of crocin and crocetin [5], is generally referred to as gardenia yellow. As an excellent natural food coloring material, gardenia yellow exhibits excellent water solubility properties and may therefore the used as a dyeing powder. Moreover, gardenia yellow is nontoxic [6] and is minimally affected by changing pH values or the presence metal metal ions. In China and Japan, the dye has received government approval to find use as a food additive and is therefore extensively applied in food industry [7]. Additionally, it has been shown to display potential pharmacological activities, e.g., antioxidant [8] and anticancer properties [9]. As such, it may be used in adjuvant therapy for the treatment of major depressive disorders [10] or reduce the side effects resulting from chemotherapy [11]. Extraction methods currently reported for gardenia yellow involve traditional solvent extraction [12], homogenate extraction [13], and ultrasound-assisted extraction [14]. The fundamental principles of these methods are based on the solubility and permeability of the corresponding solvents used. Unfortunately, unwanted species such as geniposide present in high concentration and with similar solubility may be extracted concurrently. Nevertheless homogenate or ultrasound-assisted technologies are still used to date. In solid form, geniposide exists as colorless crystals but easily hydrolyzes in the presence of microorganisms to form an aglycon. The latter species is unstable and immediately condenses with amino acids to generate a blue compound [15], which is also often used as a food colorant [16]. However, when the content of geniposide in gardenia yellow exceeds 10%, cyanine anions cleaved from geniposide may affect the hue of textile products and result in a greenish color. Meanwhile, it has also been reported that geniposide exhibits potential hepatotoxic effects. As a result, the presence of geniposide in excess not only influences the quality of gardenia yellow, but poses a health risk as well. Therefore, additional purification is inevitable and geniposide should be removed as much as possible. Common purification methods for gardenia yellow involve silica-gel column chromatography, macroporous resin adsorption [17] and clay adsorption [18]. However, generally large amounts of organic solvents and harmful reagents are used to carry out the purification. Indeed, these disadvantages are contrary to environmental protection efforts and green chemistry standards. Therefore, the development of more efficient methods for the selective extraction of gardenia yellow remains an important, albeit unmet, scientific goal.

The term mechanochemistry circumscribes the combination of mechanical and chemical phenomena on a molecular scale and includes tribology, solid state chemistry, sonochemistry, shock wave chemistry, physical chemistry *etc.* Mechanochemistry can be regarded to be the interface between chemistry and mechanical engineering [19]. The mechanisms of mechanochemical transformations are generally complex and substantially different from thermal or photochemical principles [20]. The method of ball milling is a widely-used process by which mechanical force is used to achieve chemical processing and transformations. The technique is extensively applied to the preparation of alloys and polymer materials [21,22,23], organic synthesis [24,25,26] and pollutant disposal [27,28]. Recently, mechanochemistry has been successfully expanded to the extraction of active compounds from animals and plants. For example, the extraction of water-insoluble triterpene acid from pine needles has been reported to be efficient using pure water after milling with Na_2_CO_3_ and the reported extraction yield was 1.62% [29]. After milling with Na_2_CO_3_, magnolol could be isolated along with a similar compound, honokiol. Magnolol was selectively extracted by water from *Magnolia officinalis* in good yield (11.62 ± 0.11 mg/g) [30]. The introduction of mechanochemistry into the extraction process of active plant components potentially enhances the extraction yield, facilitates extraction processes and may also prevent the extensive use of harmful organic solvents. Moreover, even selective extractions may be achieved. Based on our previous studies on gardenia yellow extraction [31] and mechanochemical extraction [32], gardenia yellow and geniposide could be selectively extracted *via* mechanochemistry, further resulting in the development of an easy separation technique with reduced environmental impact.

## 2. Results and Discussion

**Figure 1:**
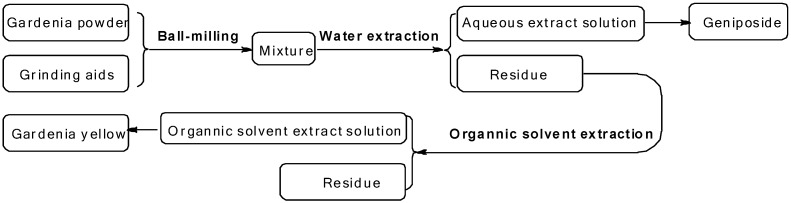
Process of the experiments.

The experimental scope required a solid system which could control the release of gardenia yellow and geniposide in different solvents. With long conjugated alkene chains, the chemical structures of crocin and its analogues contained in gardenia yellow are chemically vastly different from geniposide. Accordingly, the adsorption capacity of milling aids for those compounds varies significantly. To the best of our knowledge, amorphous solids exhibit different adsorption capacities in different solvents. Therefore, this kind of solid was chosen as the proper adjuvant and, accordingly, the solid system prepared by mechanochemistry was expected to release geniposide and gardenia yellow. The corresponding experimental process is shown in Figure 1.

### 2.1. Optimization of Ball-Milling Process

**Figure 2:**
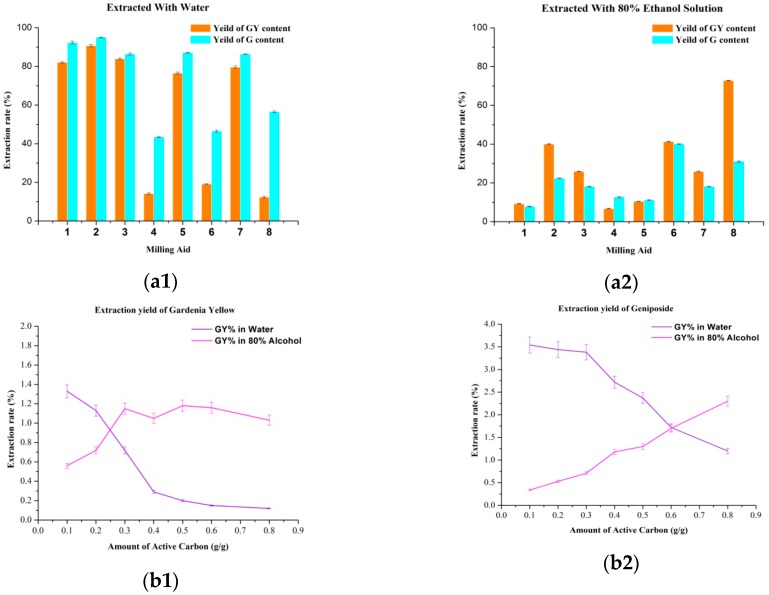
The influence of milling aids (50% wt. milling aid, 400 rpm, powder-to-ball weight ratio 1:5, 5 min) on (**a1**) Water extraction (liquid-solid ratio 20:1) at 20 °C for 5 min and (**a2**) 80% of ethanol extraction (liquid-solid ratio 20:1) at 80 °C for 5 min. 1. No additive; 2. silica gel; 3. neutral alumina; 4. zinc carbonate; 5. calcium carbonate; 6. diatomite; 7. polyaluminium chloride; 8. active carbon. The influence of different amout of activated carbon (400 rpm, powder-to-ball weight ratio 1:5, 5 min) in (**b1**)water extraction and (**b2**) alcohol extraction at the same condition.

In order to extract gardenia yellow and geniposide in a selective fashion, various water-insoluble solids with absorption capability were used as milling aids: 1. no additive; 2. silica gel; 3. neutral alumina; 4. zinc carbonate; 5. calcium carbonate; 6. diatomite; 7. polyaluminium chloride; 8. active carbon. The extraction rate of gardenia yellow and geniposide in the two-step solvent extraction was investigated after ball-milling. As shown in Figure 2, zinc carbonate, active carbon and diatomite exhibited excellent selectivity in water extraction (*cf.* Figure 2(a1)). However, gardenia yellow could not be readily released from diatomite and zinc carbonate in 80% alcohol solution (*cf.* Figure 2(a2)). Therefore, active carbon was chosen as the more appropriate milling aid, where 2.37% of the total sample weight of geniposide (56.6% of total content) were extracted by water and 1.18% of the total sample weight of gardenia yellow (72.8% of total content) were extracted using 80% ethyl alcohol.

The amount of active carbon used was further optimized to enhance both selectivity and yield. As shown in Figure 2, with increased active carbon quantity, the yields for both gardenia yellow and geniposide decreased in water extraction. Nevertheless, dependent on the absorption enhancement, the yields of both gardenia yellow and geniposide increased in 80% ethyl alcohol extraction. For the purpose of maintaining the highest selectivity, 30 wt% active carbon was finally selected (3.38% geniposide yield in water and 1.15% gardenia yellow yield in 80% ethanol).

The optimal ball mill parameters were investigated after the milling aid was fixed. For the sake of an efficient mechanochemical activation, sufficient chemical energy was supplied for the samples. In a planetary mill, mechanical energy was provided by rotation of the milling jar and was transferred to chemical energy and heat through the impact and shear force between the balls and the solid. Hence, the mechanical energy input increased directly by raising the rotation rate and by prolonging the milling time. However, when the number of balls was fixed, a higher quantity of samples (*i.e*., higher powder-to-ball weight ratio) led to lower chemical energy that was available to every particle, with lower heat-release. Thus, for gradual optimization, single-factor experiments were carried out to determine the optimal condition on rotation rate, ball-to-powder ratio and milling time.

As shown in Table 1, the results of the extraction yield of gardenia yellow and geniposide first increased and then decreased by increasing the rotation rate (*cf.* Table 1, entries 1 to 4), decreasing the powder-to-ball weight ratio (*cf.* Table 1, entries 5 to 10) and prolonging the milling time (*cf.* Table 1, entries 11 to 14), respectively. According to the principles of mechanical chemistry, when the content mass was fixed, increasing the rotation rate and prolonging the milling time would result in an increase of mechanical energy input and more energy could be provided for chemical activation and therefore improve the extraction yield. However, when the rotation rate reached 200 rpm or when the milling process lasted more than 20 min, the heat release also increased, resulting in a reduced extraction yield of gardenia yellow and geniposide. Meanwhile, a similar result was also found upon changing the powder-to-ball weight ratio. Higher or much lower powder-to-ball weight ratios result in a decreased extraction efficacy. By comparison of the experiment results, the best ball-milling conditions were determined to be: powder-to-ball weight ratio = 1:5, rotation rate = 200 rpm and milling time = 5 min.

### 2.2. Optimization of Water Extraction Process

After processing *via* mechanochemistry, the optimal conditions for the extraction of geniposide with water were investigated. Gardenia contains a large amount of pectins [33]. Therefore, the resulting extract solution would feature a very high viscosity at room temperature and such a thick pectin solution would inhibit the permeation of geniposide from the cell walls, ultimately leading to filtration loss. A higher liquid-solid ratio resulted in a lower pectin concentration and led to an improved extraction yield (*cf.* Table 2, entries 1 to 3). However, at liquid-solid ratios of 10:1 or greater, the temperature and length of extraction is irrelevant to the extraction rate, and so for maximum efficiency and minimization of energy use, the shortest extraction time (5 min) and lowest temperature (20 °C) were selected.

### 2.3. Optimization of Organic Solvent Extraction Process

**Figure 3:**
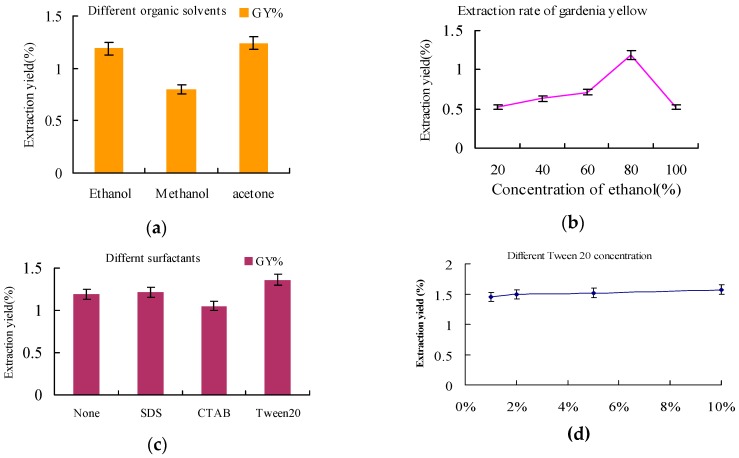
The influence of: (**a**) different 80% organic solvents (liquid-solid ratio 20:1 at its own reflux temperature for 5 min, no additive); (**b**) ethanol concetration (liquid-solid ratio 20:1 at 80 °C for 5 min, no additive); (**c**) different surfactants (80% ethanol solution, liquid-solid ratio 20:1 at 80 °C for 5 min) on yield of gardenia yellow; (**d**) different concentration of Tween 20 (80% ethanol solution, liquid-solid ratio 20:1 at 80 °C for 5 min) on yield of gardenia yellow.

To further improve the yield and selectivity, the organic solvent extraction conditions were also optimized. Both ethyl alcohol and acetone could promote desorption of gardenia yellow from active carbon and a slightly higher yield was obtained by using acetone (*cf.* Figure 3a). However, in view of environmental concerns and the small difference in performance, ethyl alcohol was selected as the desorption solvent of choice. The yield of gardenia yellow increased by raising the concentration of ethyl alcohol (*cf.* Figure 3b). However, using anhydrous ethanol resulted in a much lower yield. The use of surfactants could decrease the surface area and promote desorption. Thus, three different types of surfactants (*i.e*., CTAB: cationic surfactant, SDS: anionic surfactant, Tween 20: neutral surfactant) were added during the extraction (*cf.* Figure 3c). According to the obtained results, the neutral surfactant Tween 20 was demonstrated to be the most effective. This finding is most likely due to the higher molecular weight of Tween 20 that translates to a higher number of charged binding sites and therefore a reduced surface adhesive force of active carbon. For this reason, gardenia yellow could be rapidly desorbed from active carbon. By increasing the amount of Tween 20, the extraction yield of gardenia yellow was found to slightly increase. Tween 20 is often used as an emulsifier in food industry. However, too much additive will decrease quality of final product. Therefore, 1% Tween 20 was selected as the optimum additive concentration, improving the extraction yield of gardenia yellow at conditions that ensured high quality.

Next, basic parameters, e.g., extraction temperature, time and liquid-solid ratio, were studied. According to the results obtained, upon increasing the liquid-solid ratio, the extraction yield increased (*cf.* Table 3, entries 1 to 4). However, as the liquid-solid ratio increased more than 5:1, the extraction yield only slightly increased and the material loss in post processing increased. In order to reduce the amount of solvent used, a 5:1 liquid-solid ratio was selected. One advantage of this mechanochemical extraction method is the extraction time that exhibits less influence on the extraction yield and a high extraction yield could be attained in a very short time. As a result, 5 min was chosen to be the optimal extraction time (*cf.* Table 3, entries 1, 5 to 7). With increasing temperature, the extraction yield increased gradually, and 80 °C (reflux) was selected to be the optimal extraction temperature. Therefore, the optimal conditions of this organic extraction solution consisted of an 80% ethanol solution (liquid-solid ratio of 5:1) with addition of 1% wt Tween 20 at 80 °C for 5 min. The overall gardenia yellow yield increased to 1.45% with only 0.39% geniposide present.

### 2.4. Contrastive Analysis on Extract Methods of Gardenia Yellow

In traditional methods, no selectivity can be determined in the extraction process of gardenia yellow from gardenia fruits, no matter whether ultrasonic or enzymatic treatment techniques are applied. Unfortunately, due to its similar solubility properties no matter what solvent type is used, geniposide is always extracted together with gardenia yellow (*cf.* Table 4) [34]. However, our method is able to combine both extraction and separation. After two facile solvent extraction steps, geniposide and gardenia yellow could be extracted separately in different solvents. The entire extraction process showed higher extraction yields with the consumption of low amounts of organic solvent (*cf.* Table 4).

### 2.5. Possible Mechanism of Increasing Extraction Yield And Selective Gardenia Yellow Extraction

**Figure 4:**
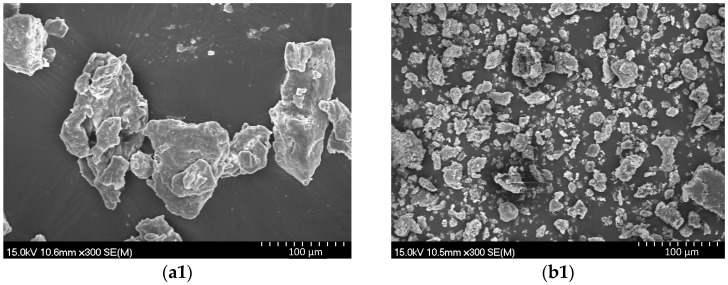

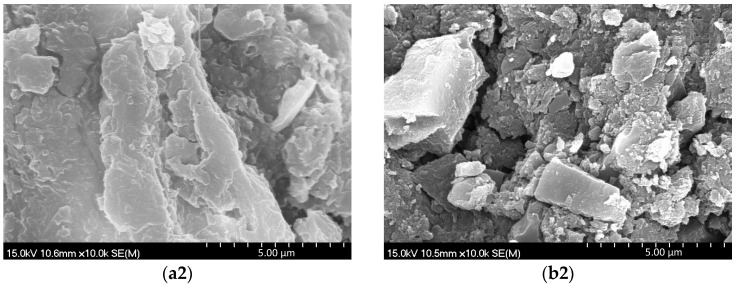
Gardenia powder at 300× magnification (**a1**) and 10,000× magnification (**a2**). Milled mixture of active carbon and gardenia (powder-to-ball weight ratio at 1:5, rotation rate at 200 rpm and milling 5 min) at 300× magnification (**b1**) and 10,000× magnification (**b2**).

The influence of mechanical force on the microstructure of gardenia and active carbon in the ball-milling process proves to be an important factor in increasing the extraction yield and selectivity. Firstly, mechanical force dramatically increases the fragmentation degree of gardenia. Furthermore, a large number of fissures appear on the surface of the gardenia powder particles (*cf.* Figure 4). As a result, solvent molecules are more likely to penetrate the cell wall and active compounds may be removed more efficiently. This results in significantly improved extraction yields, shortened extraction times, and reduced extraction temperatures.

**Figure 5:**
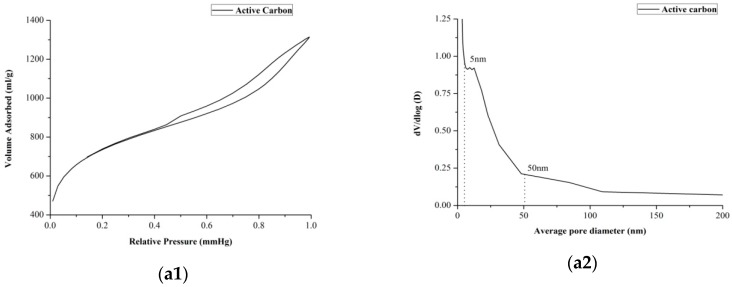

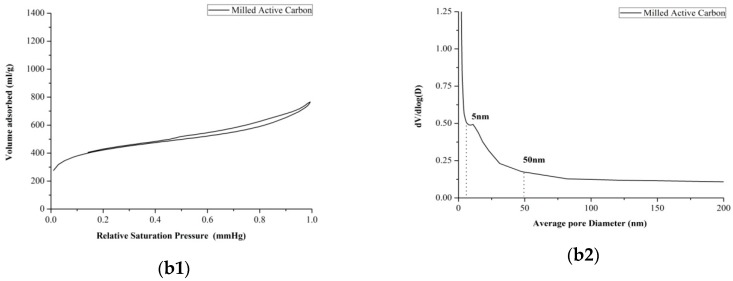
Isothermal adsorption curves of (**a1**) Active carbon. (**b1**) Milled active carbon (powder-to-ball weight ratio at 1:5, rotation rate at 200 rpm and milling 5 min) and pore size distribution curves of (**a2**) Active carbon. (**b2**) Milled active carbon (powder-to-ball weight ratio at 1:5, rotation rate at 200 rpm and milling 5 min).

To gain further insights into the mechanism of this selective extraction method, measurements of the surface area and pore distribution of untreated active carbon and milled active carbon were studied. As shown in Table 5, the BET surface area and micro-pore volume of active carbon decreased significantly and the average pore diameter increased slightly after milling. More detailed information could be obtained from isothermal adsorption curves and pore size distribution graphs before and after milling (*cf.* Figure 5). After ball milling, the surface structure of active carbon changed by mechanical force, as the volume of micropores compressed and the apertures expanded so that the adsorption capacity changed significantly.

In comparison with geniposide, gardenia yellow features a greater charge density. Hence, gardenia yellow may be more easily absorbed by active carbon than the former compound. As the adsorption capacity of active carbon decreased after ball milling, geniposide may be more easily desorbed in water. Therefore, it is assumed that mechanical force changes the structure of activated carbon, and the geniposide desorption rate increment was found to be much higher than gardenia yellow. This in turn results in a highly selective water extraction.

## 3. Materials and Methods

### 3.1. Preparation of Raw Materials

Gardenia plants treated by Chinese herbal medicine processing were purchased from the Changda Decoction Pieces Factory (Ango, Jiangxi, China). The moisture content was 91.63% ± 0.2% (g/g). The plants were harvested in JiangXi from July to August 2012 and were stored in the dark at room temperature prior to carrying out the experiments. The shape of the gardenia fruit was oval, 10 to 50 mm long and 10 to 15 mm in diameter; the fruit generally exhibits six markedly raised ridges with a calyx or scar on one end and sometimes a peduncle at the other end. The inner surface of the pericarp is yellow-brown, smooth and lustrous. The fruit can be internally divided into two loculi, containing a mass of seeds in yellow-red to dark red placente. The seeds have a bitter taste and are nearly circular, flat and the major axis is approximately 5 mm long. Based on the description and analytical approaches in Chinese Pharmacopoeia [35], the raw material was identified to be the fruit of *Gardenia jasminoides Ellis*. Before starting the experiments, the overall gardenia yellow and geniposide content was determined to be 62% ± 0.15% (g/g) and 4.19% ± 0.07% (g/g).

### 3.2. Chemicals and Reagents

α-Crocin (≥98%) and geniposide (≥98%) of analytical grade were purchased from Aladdin (Shanghai, China). HPLC grade acetonitrile was obtained from J&K Chemical Ltd. (Shanghai, China). Silica gel (100~200 mesh), Diatomite (filter aid, flux calcined, D50: 19.6 μm), neutral alumina (100 to 200 mesh), basic zinc carbonate (AR, 100 to 200 mesh), calcium carbonate (AR, 99%, 100 to 200 mesh) and activated carbon (AR, 200 mesh, brown) were obtained from Sinopharm Chemical Reagent Co. (Shanghai, China). Aluminum polychloride (PAC) (aluminum content ≥29%, basicity = 60% to 95%, pH = 3.5~5.0) was purchased from Tianjin Dingshengxin Chemical Reagent Co. (Tianjin, China). Deionized water was further purified using a Milli-Q water-purification system from Millipore (Bedford, MA, USA).

### 3.3. Ball-Milling Process

A planetary ball mill (Retsch, Haan, Germany) was used to carry out the mechanochemical extraction using the following parameters: the effective solar gear diameter was 157 mm, the speed of rotation and revolution of mill jar was 2:1. The capacity of the stainless steel milling jar was 500 mL, with a height and outer diameter of 106 mm and 106 mm, respectively. The grinding component used were steel balls (diameter 22 mm, 325 g/load). The rotational speed of the jar was set from 100 to 600 rpm and the processing time ranged from 1 to 30 min. Gardenia was primarily crushed into 40 mesh particles and was well mixed with the adjutants before performing the tests. Parallel experiments were carried out three times and an average value was reported.

### 3.4. Ultraviolet/Visible Spectrophotography Assay of Gardenia Yellow

The extraction yield of gardenia yellow (GY%) was measured using a method described by the national standard of gardenia yellow (GB 7912-2010). The absorbance of a properly diluted extract was measured at 440 nm with an ultraviolet/visible spectrophotometer (Spectrum 765PC, Shanghai Spectrum Instruments Co., Shanghai, China). The yields of gardenia yellow (GY%) were expressed as the amount of gardenia yellow extracted per gram of dried gardenia powder (weight percent, wt %). The yield was calculated according to Equation (1) and referring to the calibration curves using α-crocin as the standard. All assays were performed in triplicate.

GY% = ((ABS − I) × D × V_extract_)/(m_gardenia_ × (1 − %MC) × S × 10^6^) (1)

where:
ABSAbsorbance
SSlope factor from the standard curve (S = 0.0894)IIntercept factor from the standard curve (I = 0.0145)DDilution factor used for diluting the extracts to reach an absorbance in the range 0.4–0.7V_extract_Amount (mL) of extract solutionm_gardenia_Amount (g) of pretreated gardenia fruits used for the extraction10^6^Factor to convert the results from μg/g to g/gGY%Extraction yield of gardenia yellow from gardenia fruits (g/g)% MCMoisture content of the pretreated gardenia fruits used for extraction (%, g/g)

The color value of gardenia yellow was measured using a method described by the national standard of gardenia yellow (GB 7912-2010). The ample (0.1 g) was dissolved in deionized water (50 mL) and the absorbance was measured using an ultraviolet/visible spectrophotometer (Spectrum 765PC) at an absorption maximum of 440 nm. The color value of gardenia yellow was calculated according to Equation (2) and all assays were performed in triplicate.

(2) $$E_{1\mathrm{cm}}^{1\%}(440\;\mathrm{nm}\pm 5\;\mathrm{nm})=\mathrm{ABS}/(c\times 100)$$

where:
ABSAbsorbance
cConcentration of gardenia yellow solution (g/mL)$E_{1\mathrm{cm}}^{1\%}$ (440 nm ± 5 nm)Color Value

### 3.5. HPLC Assay of Geniposide

The amount of geniposide was determined by high performance liquid chromatography (HPLC) according to the procedures reported in the national standard of gardenia yellow (GB 7912-2010). HPLC analyses were performed on an Agilent 1200 liquid chromatography system (Agilent Technologies, Santa Clara, CA. USA), equipped with a vacuum degasser, four single solvent delivery pumps, a column compartment, a 20 μL sample loop auto injector and a diode array detector. Samples were separated on an Agilent Zorbax SB-C_18_ column (250 mm × 4.6 mm, i.d. 5 μm particle size) at a constant temperature of 40 °C. Geniposide was eluted at a flow rate of 0.7 mL/min for 20 min using an isocratic mobile phase consisting of acetonitrile and ultrapurified water at a ratio 15:85 (*v*/*v*). The diode array detector was operated at a detection wavelength of 238 nm. The eluted fractions were identified by comparing the retention times of geniposide with the corresponding standard. The concentration was calculated by integration of the peak area of geniposide referring to the calibration curves. Yields of geniposide (G%) were expressed as the amount of geniposide per gram of dried gardenia powder in weight percent (wt%) and were calculated according to Equation (3). All assays were performed in triplicates.

G% = ((PA − I) × D × Vextract)/(mgardenia × (1 − %MC) × S × 106)
(3)

where:
PAPeak Area of geniposide.SSlope from the standard curve (S = 0.0541)IIntercept from the standard curve (I = 0.225)DDilution factor used for diluting the extracts to a reach peak area in the range 100–1000.V_extract_Amount (mL) of extract solution.m_gardenia_Amount (g) of pretreated gardenia fruits used for the extraction.10^6^Factor to convert the results from μg/g to g/gG%Extraction yield of geniposide from gardenia fruits (g/g)% MCMoisture content of the pretreated gardenia fruits used for extraction (%, g/g)

### 3.6 Measurement of BET Surface Area and Observation of Microscopic Structure

The specific surface area and porosity was determined by using a physisorption analyzer (Micromeritics ASAP, Norcross, GA, USA). To measure the nitrogen BET surface area, small amounts of samples (0.25 g) were analyzed in the instrument and the nitrogen BET surface area values and pore distribution were automatically calculated by the system’s internal software. The results were reported as curves in the figures. Electron micrographs were taken on a cold field emission gun scanning electron microscope (Hitachi S-4700, Hitachinaka, Japan). The samples were measured without prior coating.

## 4. Conclusions

We have developed a new method based on mechanochemistry for the extraction and separation of two active compounds, *i.e.*, gardenia yellow and geniposide, from gardenia. According to single-factor experiments, optimal conditions for ball-milling and solvent extraction were developed as follows: gardenia powder and 30 wt% active carbon were mixed and milled in a planetary ball-mill (powder-to-ball weight ratio 1:5) at 200 rpm for 5 min. Next, the extraction was carried out in water (liquid-solid ratio 10:1) at 20 °C for 5 min. After filtration and complete removal of solvents, the remaining material was extracted with 80% ethanol solution (liquid-solid ratio 5:1) at 80 °C for 5 min. Under optimized conditions, the extraction yields for geniposide in water reached 3.59% ± 0.108% while the extraction yields of gardenia yellow in 80% ethanol solution reached 1.45% ± 0.108 g/g. The color scale for gardenia yellow extracted from 80% ethanol solution was 150 ± 5. Residual geniposide was less than 5%. Without separation, gardenia yellow with high color scale along with geniposide could be extracted separately using an alcohol solution and water. The consumption of both energy and solvent could be reduced and the entire gardenia fruit could be used. Therefore, our extraction method has been demonstrated to be both environmentally friendly and effective, potentially providing a promising alternative for the extraction of active compounds in pharmaceutical industry.

## Figures and Tables

**Table 1:** The influence of rotation rate, milling time and powder-to-ball weight ratio.

Entry	Rotation Rate (rpm)	Powder-to-Ball Weight Ratio ^a^ (g/g)	Milling Time (min)	Gardenia Yellow Extraction Rate (%) ^b^		Geniposide Extraction Rate (%) ^c^	
				Water	Alcohol	Water	Alcohol
1	100	1:5	5	0.65 ± 0.03	1.09 ± 0.03	3.06 ± 0.14	0.40 ± 0.02
2	200	1:5	5	0.76 ± 0.02	1.18 ± 0.06	3.46 ± 0.16	0.79 ± 0.03
3	400	1:5	5	0.72 ± 0.02	1.15 ± 0.05	3.38 ± 0.12	0.71 ± 0.03
4	600	1:5	5	0.52 ± 0.02	0.96 ± 0.02	3.04 ± 0.12	0.44 ± 0.02
5	200	1:1	5	0.67 ± 0.03	1.14 ± 0.03	3.04 ± 0.07	0.40 ±0.01
6	200	1:2	5	0.71 ± 0.01	1.16 ± 0.03	3.23 ± 0.15	0.57 ± 0.03
7	200	1:3	5	0.73 ± 0.03	1.20 ± 0.06	3.30 ± 0.09	0.65 ± 0.02
8	200	1:6	5	0.73 ± 0.03	1.16 ± 0.02	3.48 ± 0.17	0.80 ± 0.03
9	200	1:8	5	0.62 ± 0.03	1.06 ± 0.04	3.38 ± 0.07	0.71 ±0.03
10	200	1:10	5	0.44 ± 0.01	0.90 ± 0.04	3.29 ± 0.13	0.62 ± 0.03
11	200	1:5	1	0.73 ± 0.03	1.12 ± 0.04	2.75 ± 0.10	0.44 ± 0.01
12	200	1:5	10	0.77 ± 0.04	1.17 ± 0.03	3.50 ± 0.13	0.71 ± 0.03
13	200	1:5	20	0.76 ± 0.02	1.18 ± 0.05	3.49 ± 0.15	0.79 ± 0.03
14	200	1:5	30	0.77 ± 0.03	1.09 ± 0.04	3.49± 0.13	0.40 ± 0.01
15	Mixture without milling			0.098 ± 0.022	0.88 ± 0.02	0.112 ± 0.001	1.53 ± 0.04

^a^ Samples contained 30% activated carbon; ^b^ Water extraction (liquid-solid ratio 20:1) at 20 °C for 5 min; ^c^ 80% of ethanol extraction (liquid-solid ratio 20:1) at 80 °C for 5 min.

**Table 2:** The influence of extraction conditions on yield of geniposide in water ^a^.

Entry	Liquid-Solid Ratio (mL/g)	Temperature (°C)	Extraction Time (min)	Geniposide Extraction Rate (%)
1	5:1	20	5	2.48 ± 0.12
2	10:1	20	5	3.59 ± 0.13
3	20:1	20	5	3.50 ± 0.09
4	10:1	40	5	3.59 ± 0.18
5	10:1	60	5	3.59 ± 0.11
6	10:1	80	5	3.55 ± 0.14
7	10:1	100	5	3.57 ± 0.11
8	10:1	20	10	3.57 ± 0.08
9	10:1	20	20	3.53 ± 0.09
10	10:1	20	30	3.52 ± 0.15

^a^ Samples contained 30% activated carbon and milling condition was 200 rpm, powder-to-ball weight ratio 1:5, 5 min.

**Table 3:** The influence of extraction conditions on yield of gardenia yellow in 80% ethanol solution ^a,b^.

Entry	Liquid-Solid Ratio (mL/g)	Temperature (°C)	Extraction Time (min)	Gardenia Yellow Extraction Rate (%) ^c^
1	2:1	80	5	1.31 ± 0.04
2	5:1	80	5	1.45 ± 0.04
3	10:1	80	5	1.42 ± 0.06
4	20:1	80	5	1.36 ± 0.05
5	5:1	80	10	1.45 ± 0.05
6	5:1	80	20	1.47 ± 0.03
7	5:1	80	30	1.49 ± 0.07
8	5:1	20	5	1.13 ± 0.03
9	5:1	40	5	1.24 ± 0.05
10	5:1	60	5	1.40 ± 0.04

^a^ Samples contained 30% activated carbon and milling condition was 200 rpm, powder-to-ball weight ratio 1:5, 5 min; ^b^ Water extraction (liquid-solid ratio 10:1) was performed at 20 °C for 5 min, remaining was fully dried under 45 °C for subsequent extraction; ^c^ 1% Tween 20 was used.

**Table 4:** Comparison with different methods in gardenia yellow extraction.

Entry	Method	Solvent	Coler Value	OD	Qualified
1	Reflux	60% alcohol	187	2.72	N
2	Enzyme	Water	56	3.26	N
3	Ultrasound	60% alcohol	241	2.54	N
4	Mecanical chemistry	Water/60% alcohol	150	0.38 ± 0.055% (Geniposide content) ^a^	Y

^a^ In the eliminated version of gardenia yellow national standard, geniposide was determined at 238 nm with spectrophotometer. OD is defined as A_238_/A_440_, it should be lower than 0.4 if gardenia yellow is qualified. Now it had been replaced by a HPLC method in the current national standard and the qualification standards were changed at the same time.

**Table 5:** Specific surface area and porosity of samples.

Entry	Samples	BET Surface Area ^a^ (m^2^/g)	BJH Desorption Average Pore Diameter ^a^ (nm)	Micropore Volume ^a^ (cm^3^/g)
1	Activated carbon	2647.96	4.54	0.39
2	Milled activated carbon ^b^	1520.48	4.78	0.25

^a^ 4V/A by BET; ^b^ Mill condition. (200 rpm, powder-to-ball weight ratio 1:5, 5 min).

## Abbreviations

The following abbreviations are used in this manuscript:

GY%	Extraction rate of gardenia yellow
G%	Extraction rate of geniposide
MC	Moisture content
SEM	Scanning Electron microscope
SDS	Sodium dodecyl sulphate
CTAB	Hexadecyltrimethylammonium bromide
Tween 20	Polyoxyethylene(20)Sorbitan Monolaurate
